# Comparisons of electrophysiological characteristics, pacing parameters and mid- to long-term effects in right ventricular septal pacing, right ventricular apical pacing and left bundle branch area pacing

**DOI:** 10.1186/s12872-022-02855-8

**Published:** 2022-09-19

**Authors:** Wenhua Li, Yu Ding, Chao Gong, Genqing Zhou, Xiaofeng Lu, Yong Wei, Shi Peng, Lidong Cai, Tianyou Yuan, Fangfang Li, Shaowen Liu, Songwen Chen

**Affiliations:** 1 Department of Cardiology, Shanghai General Hospital of Nanjing Medical University, No. 100 Haining Road, Hongkou District, Shanghai, 200080 China; 2 Department of Cardiology, Wujin Hospital Affiliated with Jiangsu University, The Wujin Clinical College of Xuzhou Medical University, Changzhou City, Jiangsu Province China; 3 Department of Cardiology, Shanghai General Hospital, Shanghai Jiao Tong University School of Medicine, Shanghai, China; 4 Department of Anesthesiology, Shanghai General Hospital, Shanghai Jiao Tong University School of Medicine, Shanghai, China

**Keywords:** Electrophysiological parameters, Right ventricular septal pacing, Left bundle branch area pacing, Left ventricular activation time, Polarity of pacing parameters, Physiological pacing

## Abstract

**Background:**

As a near-physiological pacing innovation, left bundle branch area pacing (LBBAP) has drawn much attention recently. This study was aimed to investigate the electrophysiological characteristics, unipolar/bipolar pacing parameters and mid- to long-term effects and safety of three different pacing methods and identify possible predictors of adverse left ventricular remodeling.

**Methods:**

Ninety-two patients were divided into the LBBAP group, right ventricular septal pacing (RVSP) group and right ventricular apical pacing (RVAP) group. Baseline information, electrophysiological, pacing and echocardiographic parameters were collected.

**Results:**

The three pacing methods were performed with a similar high success rate. The paced QRSd was significantly different among the LBBAP, RVSP and RVAP groups (105.93 ± 15.85 ms vs. 143.63 ± 14.71 ms vs. 155.39 ± 14.17 ms, *p* < 0.01). The stimulus to left ventricular activation time (Sti-LVAT) was the shortest in the LBBAP group, followed by the RVSP and RVAP groups (72.80 ± 12.07 ms vs. 86.29 ± 8.71 ms vs. 94.14 ± 10.14 ms, *p* < 0.001). LBBAP had a significantly lower tip impedance during the procedure and 3-month follow up as compared to RVSP and RVAP (*p* < 0.001). Higher bipolar captured thresholds were observed in LBBAP during the procedure (*p* < 0.001). Compared to the baseline values, there was a greater reduction in left ventricular end-diastolic dimension (LVEDD) in the LBBAP group (*p* = 0.046) and a significant enlargement in LVEDD in the RVAP group (*p* = 0.008). Multiple regression analysis revealed that the Sti-LVAT was a significant predictor of LVEDD at 12 months post-procedure. At the 24-h post-procedure, significant elevations were observed in the cTnI levels in LBBAP (*p* < 0.001) and RVSP (*p* < 0.05). More transient RBB injury was observed in LBBAP. But no significant difference was found in cardiac composite endpoints among three groups (*p* > 0.05).

**Conclusions:**

LBBAP demonstrated a stable captured threshold, a low tip impedance and a high R-wave amplitude during the 12-month follow-up. Left ventricular remodeling was improved at 12 months post-procedure through LBBAP. The Sti-LVAT was a significant predictor of left ventricular remodeling. LBBAP demonstrated its feasibility, effectiveness, safety and some beneficial electrophysiological characteristics during this mid- to long-term follow-up, which should be confirmed by further studies.

## Introduction

Electrical pacing is an important and effective therapy for the patients with bradycardia and cardiac conduction dysfunction. Traditional right ventricular apical pacing (RVAP) causes left ventricular electrical and mechanical dyssynchrony, resulting in atrial fibrillation or heart failure (HF) [1, 2]. Alternative right ventricular outflow tract or septal pacing has not shown consistent results in improving clinical outcomes [3, 4]. Recently, physiological pacing techniques have drawn much attention.

Currently, His bundle pacing (HBP) is accepted as a real physiological pacing technology for its feasibility and clinical benefits [5, 6]. However, some limitations of HBP have been confirmed in several studies, resulting in potential adverse effects in long-term performance [7–9]. Thus, as an alternative to HBP, left bundle branch area pacing (LBBAP), was first reported by Huang et al. [10]. However, differences in electrophysiological parameters, polarity of pacing parameters and mid- to long-term effects on left ventricular function among LBBAP, right ventricular septal pacing (RVSP) and RVAP have not been thoroughly compared. Moreover, the correlations between electrophysiological parameters and left ventricular remodeling with three different pacing methods have not been systematically described. We hypothesized that some electrophysiological characteristics would reveal the prognosis of left ventricular remodeling in the mid- to long-term follow-up period after pacemaker implantation.

## Methods

### Study population

This was a retrospective cohort study of patients with indications for pacemaker implantation treatment of symptomatic bradycardia according to the established guidelines [11]. All consecutive patients from the Affiliated Wujin Hospital of Jiangsu University received implantable pacemakers between August 2017 and November 2020. A total of 142 patients were divided into the RVAP, RVSP and LBBAP groups based on the pacing site. The selection criteria for the three study groups was based on patients’ selection and operator’s personal preference and clinical practice. All recruited patients provided written informed consent. The exclusion criteria were as follows: (i) patients with a malignant tumor with a life expectancy of less than 1 year and (ii) patients less than 20 years of age or more than 85 years of age (iii) patients with myocardial infarction (MI) or hypertrophic cardiomyopathy (HCM) (iiii) patients with left ventricular ejection fraction (LVEF) ≤ 35% (iiiii) patients who were lost to follow-up.

### Implantation procedure

#### LBBAP

**Fig. 1:**
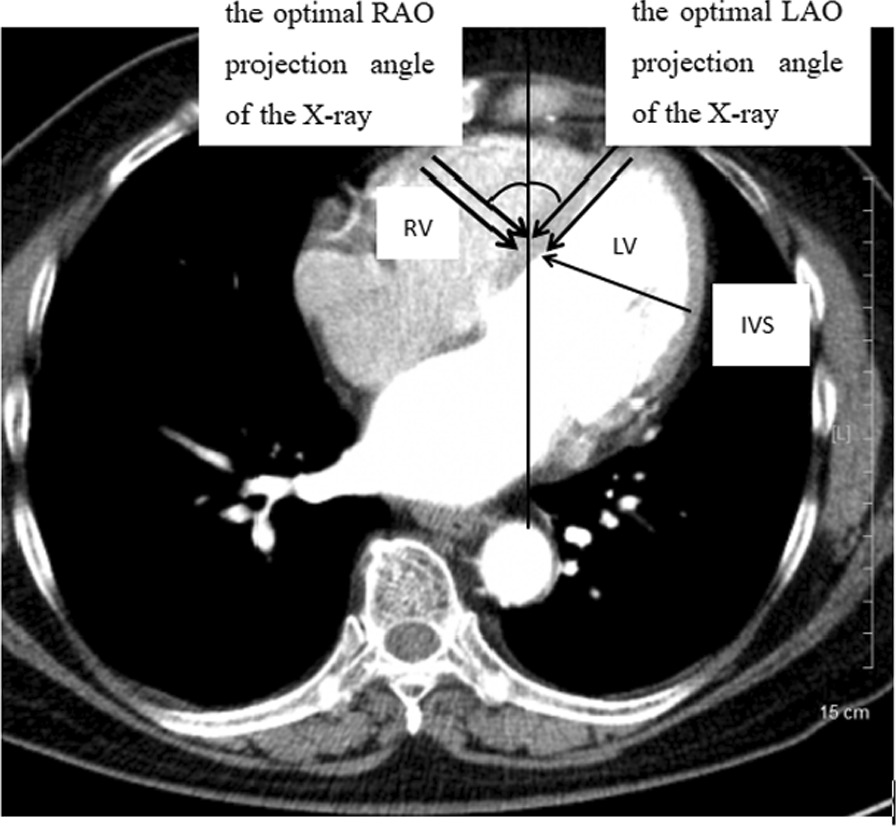
The optimal projection angle of X-ray under cardiac computerized tomography (CT). *RAO* right anterior oblique; *LAO* left anterior oblique; *RV* right ventricle; *LV* left ventricle; *IVS* interventricular septum

**Fig. 2:**
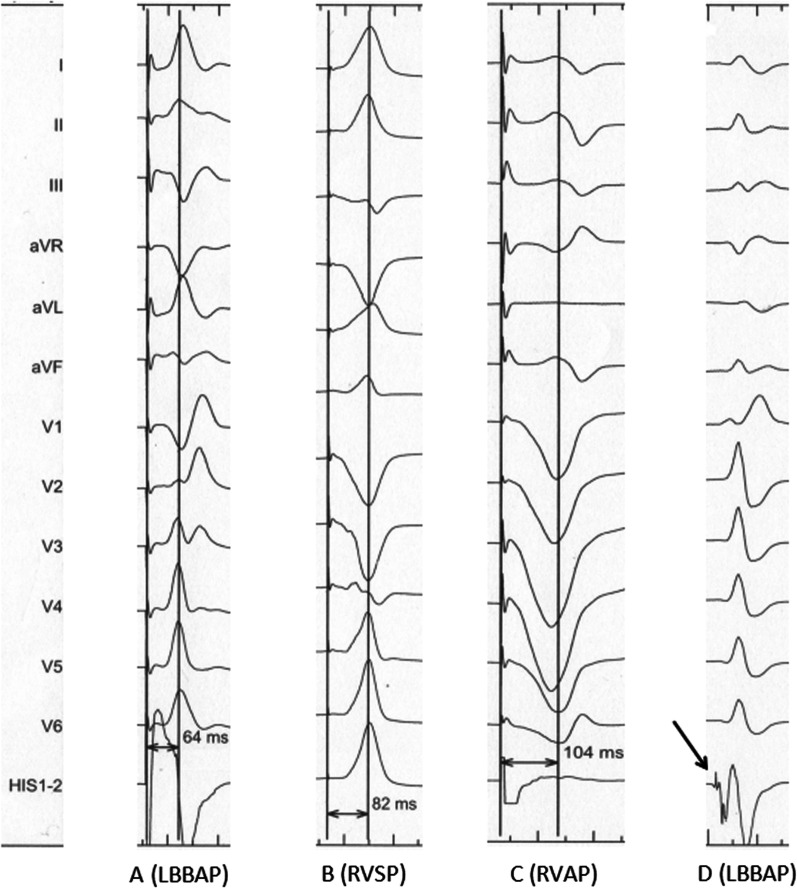
Sti-LVAT of the three pacing positions during lead implantation. (**A**) LBBAP with an Sti-LVAT of 64 ms; (**B**) RVSP with an Sti-LVAT of 82 ms; (**C**) RVAP with an Sti-LVAT of 104 ms; (**D**) A recorded LBB potential of LBBAP during lead implantation. Sti-LVAT, stimulus to left ventricular activation time; LBBAP, left bundle branch area pacing; LBB, left bundle branch; RVSP, right ventricular septal pacing; RVAP, right ventricular apical pacing

LBBAP was achieved using the 3830 lead and the C315 sheath (Medtronic, Inc., Minneapolis, Minnesota) by the transventricular-septal method as previously described [12]. In our study, cardiac computerized tomography (CT) was innovatively used to determine the optimal projection angle of the X-ray (Fig. 1). In brief, the lowest transverse plane of the aortic sinus is close to the apex of tricuspid valve annulus and the His bundle. Most studies showed that 3830 pacing lead was perpendicular to the ventricular septum and located approximately 15–20 mm below the His bundle under fluoroscopic image. In our study, the cardiac transverse plane 15–20 mm below the lowest plane of the aortic sinus was used as a reference plane. In this plane, the thickness of ventricular septum was measured accurately, which helped to estimate the depth of 3830 pacing lead into the septum before implantation. Moreover, the optimal right anterior oblique (RAO) and left anterior oblique (LAO) projection angle of the X-ray can be calculated accurately, which helped to ensure that the 3830 pacing lead was truly perpendicular to the septum (Fig. 1). Contrast injection from the sheath was used to measure the lead depth inside the septum and identify the possible perforation. The Purkinje (P) potential was often observed in most cases (Fig. 2D), and V_4̴-6_ on the surface 12-lead electrocardiogram (ECG) showed the shortest and most constant left ventricular activation time (LVAT) (Fig. 2A). The LBBAP was immediately switched to the RVSP or the RVAP when a maximum of five attempts failed.

#### RVSP

The 5076 pacing lead (Medtronic Inc. USA) was implanted at the mid-RV septum as previously described [13, 14]. Likewise, the RVSP was immediately switched to the RVAP when a maximum of five attempts failed.

#### RVAP

The 4574 pacing lead (Medtronic Inc. USA) was routinely implanted at the RV apex in a standard fashion.

### Programming

Considering the intrinsic bundle branch block and atrioventricular (AV) conduction time, AV delay programming was individualized as previously described [15].

### Definition of the stimulus to left ventricular activation time (Sti-LVAT)

Here, LVAT indicates the rapidity of left ventricular free wall activation. The short LVAT (< 80 ms) suggested capture of the conduction system. We defined the Sti-LVAT of the LBBAP or RVSP as the duration from the stimulus artifact to the peak of the R wave and the Sti-LVAT of the RVAP as the duration from the stimulus artifact to the trough of the R wave in lead V5/6 (Fig. 2A, B, C).

### Data collection and follow up

The clinical baseline characteristics were collected. Echocardiography was performed by two experienced echocardiologists. The electrophysiological and echocardiographic parameter data were collected, including the paced QRS duration (QRSd), paced QRS axis, Sti-LVAT, left ventricular end-diastolic dimension (LVEDD), left atrial diameter (LAD) and left ventricular ejection fraction (LVEF). The unipolar (tip) and bipolar pacing parameters were measured during the procedure. Patients were followed at 1 week, 1 month, 3 months and 12 months post-procedure. The mid-term follow-up was defined as 3–6 months and the long-term follow-up as more than 6 months. The echocardiographic parameters and pacing parameters were recorded at outpatient visit. The levels of cardiac troponin I (cTnI) and plasma N-terminal pro-B type natriuretic peptide (NT-pro-BNP) were recorded and evaluated at baseline and each follow-up.

### Statistical analysis

Data were analyzed using SPSS 22.0 (Chicago, IL, USA). Continuous data are reported as the means ± standard deviations (SDs) or median (interquartile ranges). Categorical data are expressed as numbers and percentages and were compared by χ2 test or Fisher exact test. An analysis of variance (ANOVA) and Tukey’s post hoc test were performed for multiple comparisons. Repeated-measures ANOVA was applied to compare the mean values of variables before the procedure, during implantation, and at each follow-up. A linear regression analysis was done in all cases. Stepwise multiple regression analysis was used to determine which variable provided the best estimate for heart function. A two-sided *p* value < 0.05 was considered significant.

## Results

### Clinical baseline characteristics

**Fig. 3:**
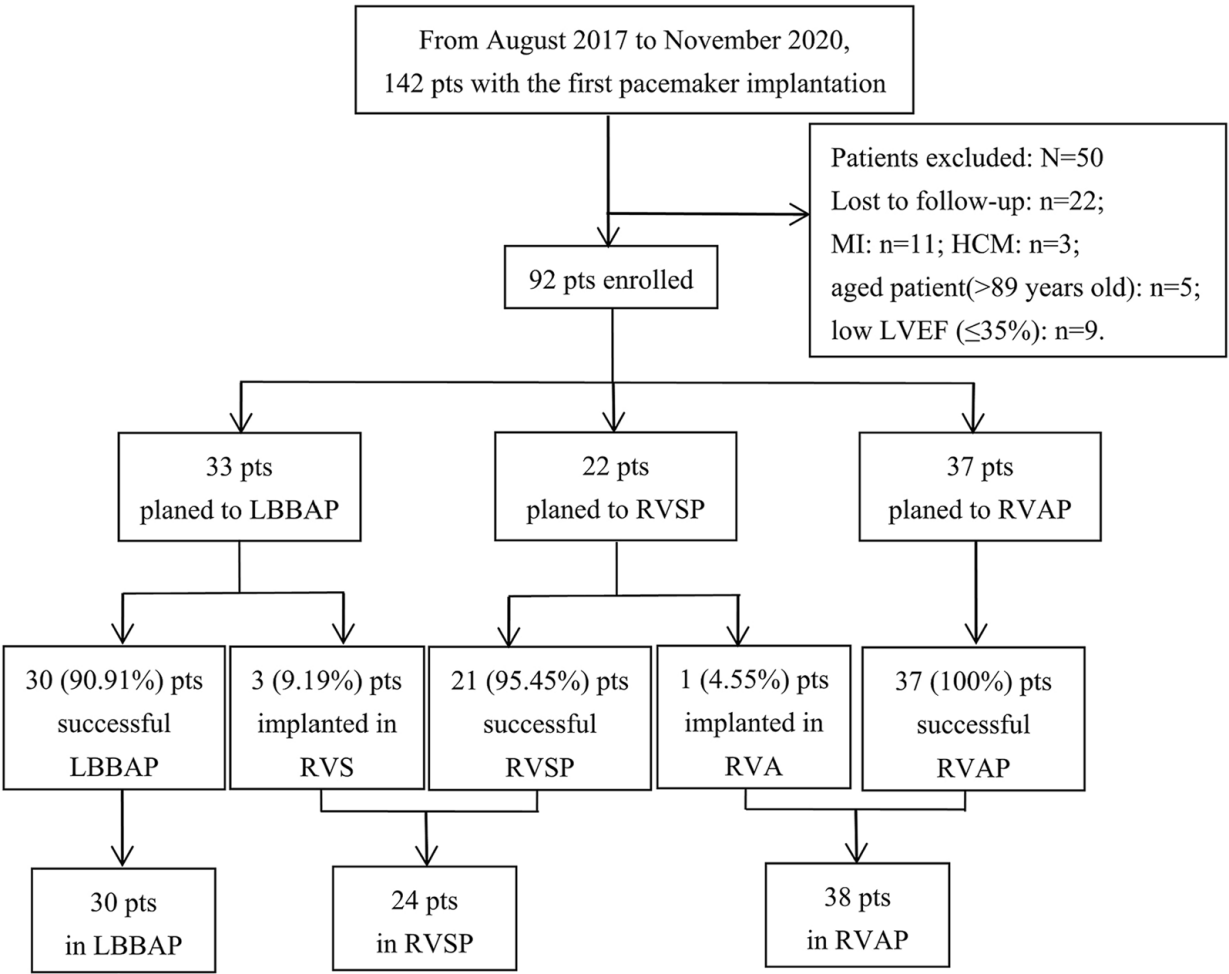
Schematic plot of the study. *Pts* patients; *MI* myocardial infarction; *HCM* hypertrophic cardiomyopathy; *LVEF* left ventricular ejection fraction; *LBBAP* left bundle branch area pacing; *RVS* right ventricular septum; *RVSP* right ventricular septal pacing; *RVAP* right ventricular apex pacing

A total of 142 patients underwent the first pacemaker implantation from August 2017 to November 2020. After the exclusion of 22 patients who were lost to follow-up, 5 patients more than 89 years of age, 9 patients with LVEF ≤ 35%, 11 patients with MI and 3 patients with HCM, 92 consecutive patients were enrolled in the study. In the LBBAP group, LBBAP was successfully achieved in 30 patients (90.91%) and the remaining 3 patients received RVSP instead due to an inability to screw the lead deep enough to reach the left bundle branch area. In the RVSP group, RVSP was successfully performed in 21 patients (95.45%), and the remaining 1 patient received RVAP due to the high pacing threshold in the RV septum. In the RVAP group, RVAP was successfully performed in 37 patients (100%). The study flowchart is presented in Fig. 3.

At baseline, the LBBAP group had more patients characterized by the LBBB pattern than the RVSP group and the RVAP group (30% vs. 16.67% vs. 5.26%, *p* = 0.021). The clinical baseline characteristics are summarized in Table 1.

**Table 1:** Comparison of clinical baseline characteristics

	LBBAP (n = 30)	RVSP (n = 24)	RVAP (n = 38)	*p*
Age (years)	70.23 ± 9.58	74.71 ± 9.36	73.13 ± 6.62	0.139
Males, n (%)	12 (40.0%)	12 (50%)	26 (68.42%)	0.068
Hypertension, n (%)	20 (66.67%)	20 (83.33%)	29 (76.32%)	0.346
Diabetes, n (%)	11 (36.67%)	6 (25.0%)	5 (13.16%)	0.076
CAD, n (%)	3 (10.0%)	4 (16.67%)	2 (5.26%)	0.370
CKD, n (%)	7 (23.33%)	3 (12.5%)	7 (18.42%)	0.625
Hyperlipidemia, n (%)	8 (26.67%)	3 (12.5%)	7 (18.42%)	0.451
Stroke, n (%)	3 (10.0%)	2 (8.33%)	4 (10.53%)	1.000
*ECG*				
AVB, n (%)	19 (63.3%)	18 (75%)	28 (73.7%)	0.571
SSS, n (%)	4 (13.3%)	5 (20.8%)	8 (21.1%)	0.745
AF with low ventricular rate, n (%)	7 (23.3%)	2 (8.33%)	2 (5.3%)	0.055
Intrinsic QRSd (ms)	122.67 ± 33.19	112.83 ± 27.23	110.39 ± 25.86	0.204
Wide QRS complex, n (%)	14 (46.67%)	11 (45.83%)	14 (36.84%)	0.666
LBBB, n (%)	9 (30%)	4 (16.67%)	2 ( 5.26%)	0.021
RBBB, n (%)	5 (16.67%)	7 (29.17%)	12 (31.58%)	0.393
*Type of pacemaker*				
single-chamber, n (%)	7 (23.33%)	3 (12.50%)	4 (10.53%)	0.373
dual-chamber, n (%)	23 (76.67%)	21 (87.50%)	34 (89.47%)	0.373
*Echocardiogram*				
LVEDD (mm)	50.73 ± 6.54	48.04 ± 4.26	49.37 ± 4.39	0.167
LAD (mm)	41.67 ± 6.41	39.46 ± 4.70	40.0 ± 5.29	0.298
LVEF (%)	56.40 ± 14.26	59.58 ± 8.44	61.68 ± 7.62	0.122
cTnI [M (P_25_, P_75_)] (ng/ml)	0.012 (0.012, 0.018)	0.014 (0.012, 0.041)	0.012 (0.012, 0.041)	0.083
NT-pro-BNP [M (P_25_, P_75_)] (pg/ml)	1140.00 (331.50, 3395.00)	1035 (234.75, 2117.50)	666.00 (313.75, 2252.50)	0.793

*CAD* coronary artery disease, *CKD* chronic kidney disease, *ECG* electrocardiograph, *AVB* atrioventricular conduction block, *SSS* sick sinus node syndrome, *AF* atrial fibrillation, *QRSd* QRS duration, *LBBB* left bundle branch block, *RBBB* right bundle branch block, *LVEDD* left ventricular end-diastolic dimension, *LAD* left atrial diameter, *LVEF* left ventricular ejection fraction, *cTnI* cardiac troponin I, *NT-pro-BNP* N-terminal pro-B type natriuretic peptide, *LBBAP* left bundle branch area pacing, *RVSP* right ventricular septal pacing, *RVAP* right ventricular apex pacing. Values are the mean (SD) or number (%). A *p* value < 0.05 was considered statistically significant

### Electrophysiological parameters and implantation results during the procedure

In the LBBAP group, the paced ECG morphology was characterized by an RBBB pattern and a narrow QRS complex. In the RVSP group and RVAP group, the paced ECG morphology showed wide QRS and LBBB patterns. The paced QRSd was significantly different among the LBBAP, RVSP and RVAP groups (105.93 ± 15.85 ms vs. 143.63 ± 14.71 ms vs. 155.39 ± 14.17 ms, *p* < 0.01). Compared with intrinsic QRSd, the paced QRSd was significantly narrower in the LBBAP group (105.93 ± 15.85 ms vs. 122.67 ± 33.19 ms, *p* < 0.01), and significantly wider in the RVSP group (143.63 ± 14.71 ms vs. 112.83 ± 27.23 ms, *p* < 0.01) and the RVAP group (155.39 ± 14.17 ms vs. 110.39 ± 25.86 ms, *p* < 0.01). In the LBBAP group, left bundle branch (LBB) potential was recorded in 21 (70.0%) patients, and the current of injury (COI) was recorded in 7 (23.3%) patients. The incidence of selective LBBP in LBBAP was 53.3%. The Sti-LVAT among the three groups was significantly different (72.80 ± 12.07 ms vs. 86.29 ± 8.71 ms vs. 94.14 ± 10.14 ms, *p* < 0.001). Compared to the RVSP group, tip captured thresholds of the ventricular lead in the LBBAP group were significantly increased (0.88 ± 0.33 V vs. 0.73 ± 0.23 V, *p* = 0.027). LBBAP had significantly high bipolar captured thresholds, followed by those in RVAP and RVSP (*p* < 0.001). In contrast, LBBAP had significantly low tip impedance, followed by those in RVSP and RVAP (*p* < 0.001). Intraprocedural parameters among the three groups are summarized in Table 2.

**Table 2:** Comparison of intraprocedural parameters among the three groups

	LBBAP (n = 30)	RVSP (n = 24)	RVAP (n = 38)	*p*
Paced QRSd (ms)	105.93 ± 15.85	143.63 ± 14.71	155.39 ± 14.17	< 0.001***
LBB potential, n (%)	21 (70%)	NC	NC	–
COI, n (%)	7 (23.3%)	NC	NC	–
Selective LBBP	16 (53.3%)	NC	NC	–
Sti-LVAT (ms)	72.80 ± 12.07	86.29 ± 8.71	94.14 ± 10.14	< 0.001***
*Paced QRS axis*				
Normal, n (%)	17 (56.67%)	10 (41.67%)	1 (2.63%)	< 0.001***
Left axis deviation, n (%)	13 (43.33%)	12 (50.0%)	37 (97.37%)	< 0.001***
Right axis deviation, n (%)	0	2 (8.33%)	0	0.066
*Pacing parameters*				
Unipolar captured thresholds (V)	0.88 ± 0.33	0.73 ± 0.23	0.77 ± 0.19	0.060
Bipolar captured thresholds (V)	0.80 ± 0.32	0.52 ± 0.13	0.56 ± 0.16	< 0.001***
Tip R-wave amplitudes (mV)	10.93 ± 5.74	15.58 ± 7.64	12.09 ± 3.63	0.057
Bipolar R-wave amplitudes (mV)	13.13 ± 6.17	13.68 ± 6.62	10.88 ± 3.68	0.072
Tip impedance (Ω)	679.13 ± 207.31	787.25 ± 187.88	883.58 ± 129.36	< 0.001***
Bipolar impedance (Ω)	708.83 ± 171.24	680.00 ± 188.21	850.13 ± 161.39	< 0.001***
*Implantation results*				
Procedure time (min)	158.0 ± 48.98	152.08 ± 55.64	111.42 ± 59.53	0.001**
Fluoroscopy time (min)	9.13 ± 2.89	7.25 ± 2.42	4.47 ± 1.98	< 0.001***
X-ray dose (mGy)	79.13 ± 26.73	69.96 ± 29.90	49.53 ± 29.78	< 0.001***

*LBB* left bundle branch, *COI* current of injury, *Sti-LVAT* stimulus to left ventricular activation time. *p values < 0.05, **p values < 0.01, ***p values < 0.001

### Echocardiographic parameters follow-up

**Fig. 4:**
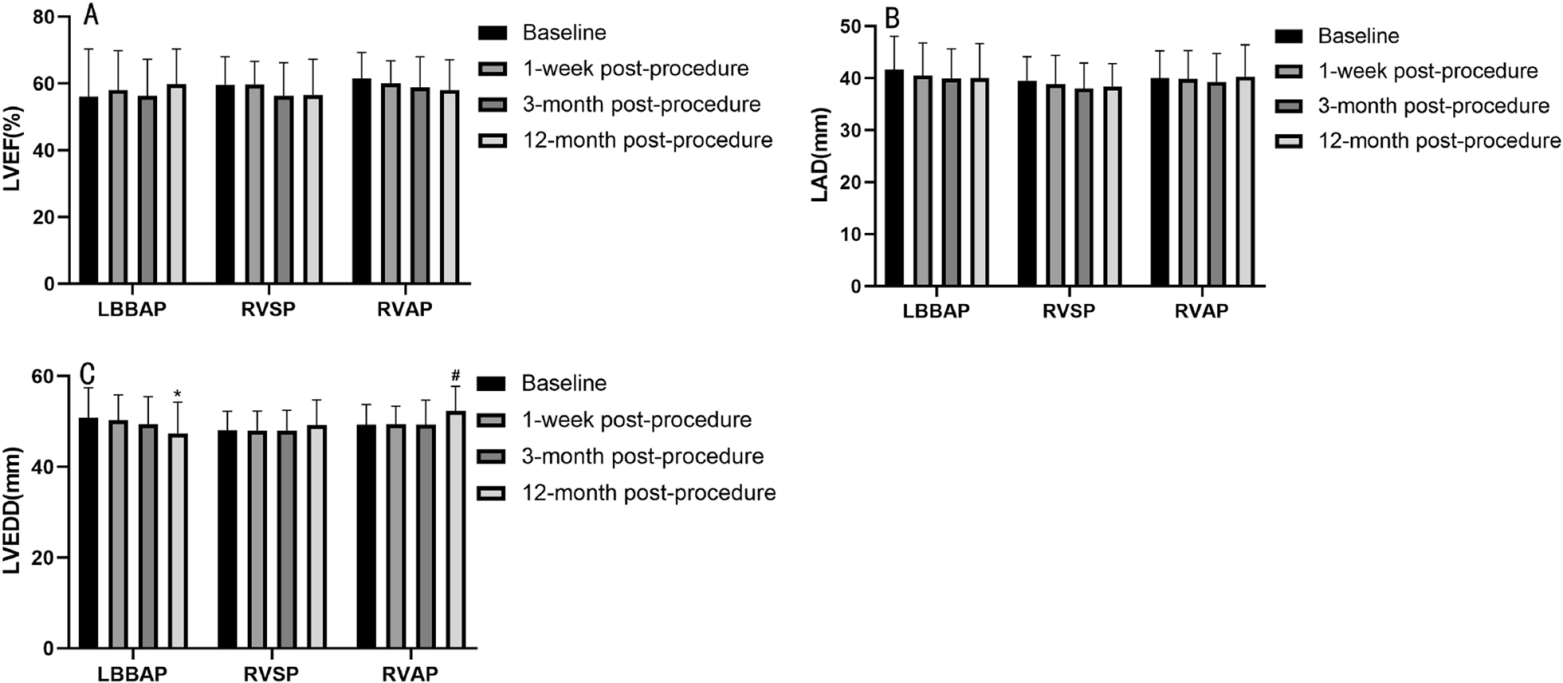
Echocardiographic parameters between baseline and each follow-up in the three groups. (**A**) Comparison of LVEF between baseline and each follow-up in the different group. (**B**) Comparison of LAD between baseline and each follow-up in the different group. (**C**) Comparison of LVEDD between baseline and each follow-up in the different group. *LBBAP* left bundle branch area pacing; *RVSP* right ventricular septal pacing; *RVAP* right ventricular apical pacing; *LVEF* left ventricular ejection fraction; *LAD* left atrial diameter; *LVEDD* left ventricular end-diastolic dimension. *p values < 0.05, ^#^*p* values < 0.01

Compared with baseline, no significant change was observed in LVEF and LAD in the LBBAP group, the RVAP group and the RVSP group during the 12-month follow-up (Fig. 4A, B). Compared to baseline, a greater reduction in LVEDD was observed at 12 months post-procedure in the LBBAP group (47.39 ± 6.95 mm vs. 50.73 ± 6.54 mm, *p* = 0.046). On the contrary, a significant enlargement in LVEDD was observed at 12 months post-procedure in the RVAP group when compared with baseline (52.37 ± 5.39 mm vs. 49.37 ± 4.39 mm, *p* = 0.008), 1 week post-procedure (52.37 ± 5.39 mm vs. 49.42 ± 4.00 mm, *p* = 0.009) and 3 months post-procedure (52.37 ± 5.39 mm vs. 49.36 ± 5.36 mm, *p* = 0.008) (Fig. 4C).

There was no significant difference in echocardiographic parameters (LVEDD, LAD, and LVEF) among the three groups at baseline (Table 1), 1 week and 3 months post-procedure (Table 3). However, a significant difference was noted in the LVEDD among the LBBAP, RVSP and RVAP groups at 12 months post-procedure (47.39 ± 6.95 mm vs. 49.23 ± 5.55 mm vs. 52.37 ± 5.39 mm, *p* = 0.004) (Table 3).

**Table 3:** Echocardiographic parameters in the three groups during the follow-up

	LBBAP (n = 30)	RVSP (n = 24)	RVAP (n = 38)	*p*
*1 week post-procedure*	*n = 30*	*n = 22*	*n = 36*	
LVEDD (mm)	50.33 ± 5.62	47.95 ± 4.38	49.42 ± 4.00	0.202
LAD (mm)	40.57 ± 6.18	38.86 ± 5.55	39.83 ± 5.49	0.575
LVEF (%)	58.00 ± 11.87	59.73 ± 6.92	60.11 ± 6.70	0.608
*3 months post-procedure*	*n = 26*	*n = 23*	*n = 36*	
LVEDD (mm)	49.46 ± 6.03	47.91 ± 4.60	49.36 ± 5.36	0.528
LAD (mm)	39.92 ± 5.72	38.00 ± 4.93	39.31 ± 5.45	0.452
LVEF (%)	56.31 ± 11.02	56.35 ± 9.92	58.92 ± 9.12	0.498
*12 months post-procedure*	*n = 28*	*n = 22*	*n = 38*	
LVEDD (mm)	47.39 ± 6.95	49.23 ± 5.55	52.37 ± 5.39	0.004**
LAD (mm)	40.04 ± 6.61	38.41 ± 4.46	40.32 ± 6.11	0.466
LVEF (%)	59.75 ± 10.64	56.64 ± 10.63	58.05 ± 9.14	0.547
ΔLVEDD (mm)	-3.21 ± 4.06	0.77 ± 4.36	3.00 ± 4.58	< 0.001***

*LVEDD* left ventricular end-diastolic dimension, *LAD* left atrial diameter, *LVEF* left ventricular ejection fraction, *ΔLVEDD* the change in LVEDD from baseline to 12-month follow-up over time. *p values < 0.05, **p values < 0.01, ***p values < 0.001

Encouragingly, there was a significant difference in changes in LVEDD (ΔLVEDD) from baseline to 12-month follow-up over time among the three groups (LBBAP, RVSP, RVAP) (−3.21 ± 4.06 mm vs. 0.77 ± 4.36 mm vs.3.00 ± 4.58 mm, *p* < 0.001) (Table 3). Compared with RVSP and RVAP, a significant reduction in LVEDD was observed in LBBAP from baseline to 12-month follow-up (−3.21 ± 4.06 mm vs. 0.77 ± 4.06 mm, *p* = 0.006; −3.21 ± 4.06 mm vs. 3.00 ± 4.58 mm, *p* < 0.001). However, the difference was not significant in ΔLVEDD between RVSP and RVAP (*p* = 0.181).

### cTnI levels and NT-pro-BNP values at baseline and at each follow-up

**Fig. 5:**
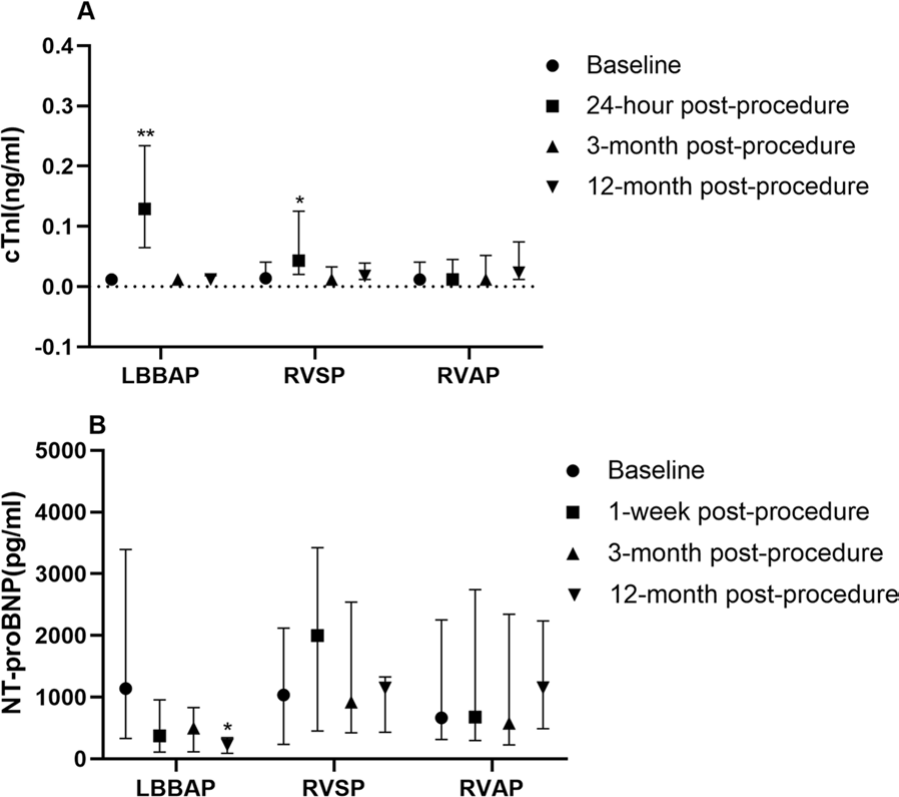
Comparisons of cTnI (**A**) and NT-proBNP values (**B**) between baseline and each follow-up for the three groups. *cTnI* cardiac troponin I; *NT-proBNP* N-terminal pro-B type natriuretic peptide. *LBBAP* left bundle branch area pacing; *RVSP* right ventricular septal pacing; *RVAP* right ventricular apical pacing. *p values < 0.05, **p values < 0.01

In comparison to baseline, the cTnI levels at 24 h post-procedure significantly increased in the LBBAP group [0.129 (0.065, 0.234) ng/ml vs. 0.012 (0.012, 0.018) ng/ml, *p* < 0.001], and the RVSP group [0.043 (0.020, 0.125) ng/ml vs. 0.014 (0.012, 0.041) ng/ml, *p* = 0.014], and remained stable in the RVAP group [0.012 (0.012, 0.045) ng/ml vs. 0.012 (0.012, 0.041) ng/ml, *p* = 0.557] (Fig. 5A). Moreover, the cTnI levels in the LBBAP group were significantly higher than those in the RVSP group at 24 h post-procedure (*p* = 0.009).

Compared with baseline, the changes in NT-proBNP values were not significant in RVAP and RVSP during 12 months follow-up period (*p* > 0.05). However, the levels of NT-pro-BNP were significantly lower in the LBBAP group at 12 months post-procedure [227.50 (90.00, 345.75) pg/ml vs. 1140.00 (331.50, 3395.00) pg/ml, *p* = 0.001] (Fig. 5B).

### Pacing parameters follow-up

At 1 week and 3 months post-procedure, the LBBAP group had a significantly lower tip impedance than the RVSP and RVAP groups (*p* < 0.001), which was maintained at 12 months post-procedure when compared with the RVAP group (*p* = 0.049). However, the difference was not significant in the tip impedance between the RVSP and RVAP groups or in tip/bipolar captured thresholds, R-wave amplitude and bipolar impedance among the three groups during the 12-month follow-up (*p* > 0.05). There was no significant difference in the average percentage of ventricular pacing among the three groups during the 12-month follow-up (*p* > 0.05). The pacing parameters during the follow-up period are summarized in Table 4.

**Table 4:** The pacing parameters during the 12-month follow-up

	LBBAP (n = 30)	RVSP (n = 24)	RVAP (n = 38)	*p*
*1 week post-procedure*	*n = 30*	*n = 23*	*n = 38*	
Tip captured threshold (V)	0.70 ± 0.22	0.66 ± 0.15	0.71 ± 0.21	0.639
Bipolar captured threshold (V)	0.64 ± 0.20	0.57 ± 0.12	0.59 ± 0.15	0.246
Tip R-wave amplitudes (mV)	15.08 ± 8.30	16.91 ± 8.42	14.82 ± 5.22	0.568
Bipolar R-wave amplitude (mV)	16.57 ± 7.58	17.91 ± 8.01	15.05 ± 6.13	0.309
Tip impedance (Ω)	558.90 ± 105.78	810.74 ± 164.54	815.55 ± 121.20	< 0.001***
Bipolar impedance (Ω)	633.23 ± 89.35	637.35 ± 125.33	687.39 ± 117.69	0.091
Percentage of ventricular pacing (%)	91.00 ± 11.83	87.70 ± 16.44	82.96 ± 19.59	0.193
*3 months post-procedure*	*n = 27*	*n = 21*	*n = 36*	
Tip captured threshold (V)	0.70 ± 0.17	0.66 ± 0.13	0.65 ± 0.18	0.555
Bipolar captured threshold (V)	0.65 ± 0.16	0.67 ± 0.14	0.60 ± 0.18	0.310
Tip R-wave amplitude (mV)	15.81 ± 8.35	17.84 ± 8.35	15.34 ± 4.97	0.471
Bipolar R-wave amplitude (mV)	17.58 ± 8.31	18.06 ± 8.23	15.34 ± .4.98	0.253
Tip impedance (Ω)	512.78 ± 98.47	704.48 ± 122.44	746.61 ± 105.36	< 0.001***
Bipolar impedance (Ω)	608.19 ± 65.35	630.00 ± 94.83	606.78 ± 88.89	0.623
Percentage of ventricular pacing (%)	85.68 ± 14.08	79.62 ± 20.81	85.87 ± 14.01	0.326
*12 months post-procedure*	*n = 27*	*n = 23*	*n = 34*	
Tip captured threshold (V)	0.80 ± 0.19	0.70 ± 0.16	0.74 ± 0.24	0.222
Bipolar captured threshold (V)	0.76 ± 0.20	0.68 ± 0.15	0.69 ± 0.21	0.245
Tip R-wave amplitude (mV)	16.88 ± 7.47	17.67 ± 7.67	15.30 ± 4.89	0.354
Bipolar R-wave amplitude (mV)	18.71 ± 7.18	17.86 ± 7.56	15.46 ± 4.66	0.096
Tip impedance (Ω)	576.96 ± 94.68	631.48 ± 99.29	651.28 ± 188.55	0.124
Bipolar impedance (Ω)	624.93 ± 80.65	606.17 ± 90.31	579.82 ± 77.06	0.103
Percentage of ventricular pacing (%)	83.21 ± 15.26	78.68 ± 18.33	83.44 ± 14.88	0.495

*p values < 0.05, **p values < 0.01, ***p values < 0.001

### Correlation between the Sti-LVAT and left ventricular function

**Fig. 6:**
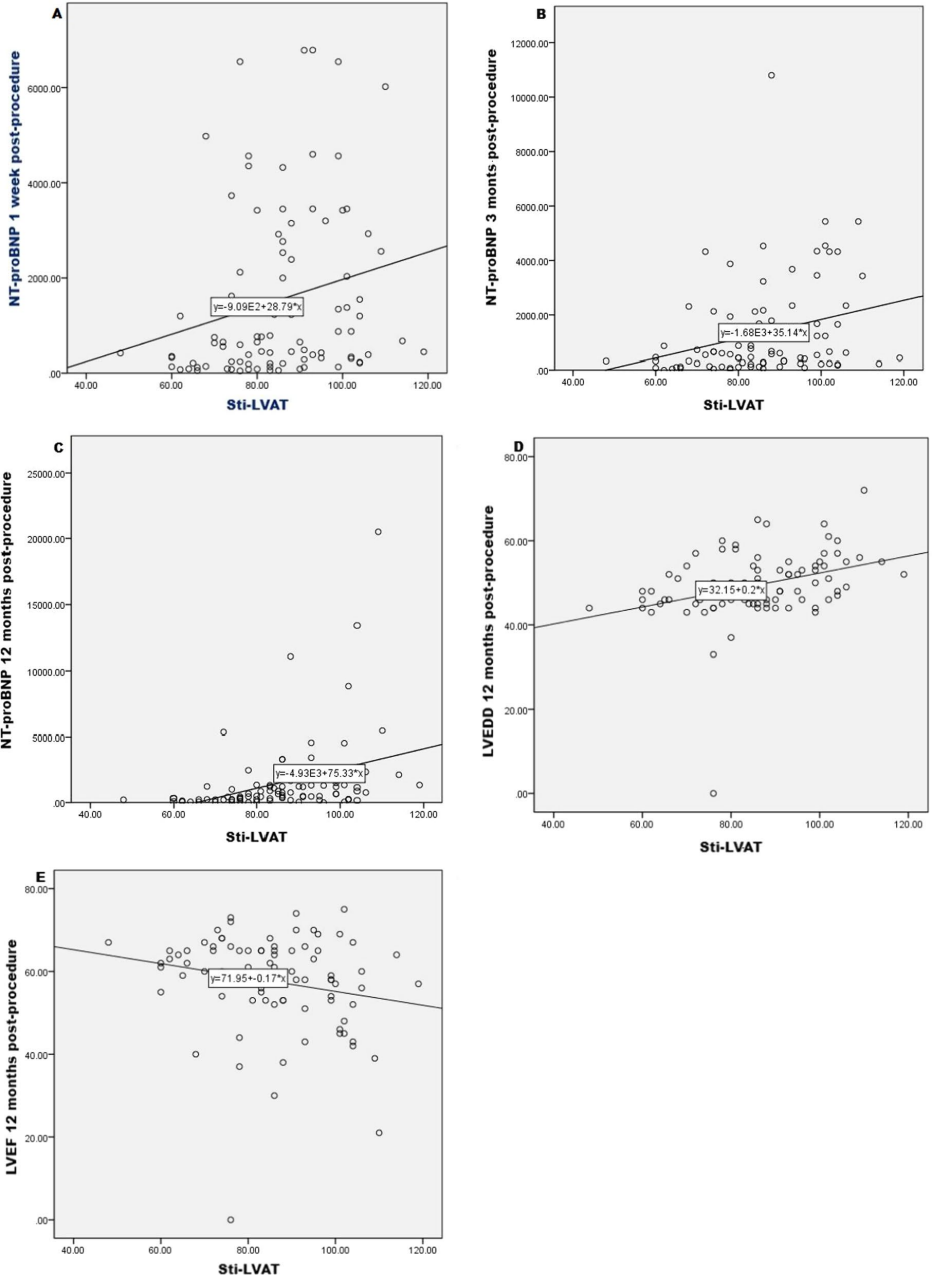
Scatter plot of correlation between the Sti-LVAT and left ventricular function. (**A**) Scatter plot of a positive correlation between the Sti-LVAT and NT-proBNP 1 week post-procedure. (**B**) Scatter plot of a positive correlation between the Sti-LVAT and NT-proBNP 3 months post-procedure. (**C**) Scatter plot of a positive correlation between the Sti-LVAT and NT-proBNP 12 months post-procedure. (**D**) Scatter plot of a positive correlation between the Sti-LVAT and LVEDD 12 months post-procedure. (**E**) Scatter plot of a negative correlation between the Sti-LVAT and LVEF 12 months post-procedure. *Sti-LVAT* the stimulus to left ventricular activation time; *NT-pro-BNP* N-terminal pro-B type natriuretic peptide; *LVEDD* left ventricular end-diastolic dimension; *LVEF* left ventricular ejection fraction

The Sti-LVAT during the procedure was related to the NT-pro-BNP levels at 1 week post-procedure (r_s_ = 0.313, *p* = 0.003), 3 months post-procedure (r_s_ = 0.309, *p* = 0.004), and at 12 months post-procedure (r_s_ = 0.516, *p* < 0.001) (Fig. 6A–C). The Sti-LVAT during the procedure was also correlated with the LVEDD and the LVEF at 12 months post-procedure (r = 0.395; 0.285, *p* < 0.001; *p* = 0.007) (Fig. 6D, E).

Linear bivariate analysis also showed positive correlation between the LVEDD at 12 months post-procedure and age (r = 0.221, *p* = 0.038), NT-pro-BNP preprocedure (r_s_ = 0.252, *p* = 0.018) and QRSd preprocedure (r = 0.258, *p* = 0.015). Stepwise multiple regression analysis, using the LVEDD at 12 months post-procedure as the dependent variable, and age, Sti-LVAT, NT-pro-BNP and QRSd preprocedure as independent variables in a predictive model, showed that only Sti-LVAT (t = 3.432, *p* = 0.001) was a significant predictor of the LVEDD (Table 5).

**Table 5:** Stepwise multiple regression analysis showing the association of LVEDD with Sti-LVAT, age, NT-pro-BNP and QRSd in a predictive model

Model	Predictors	R^2^	Adjusted R^2^	B	SE	t	*p*
1	Sti-LVAT	0.156	0.146	0.175	0.044	3.986	< 0.001***
2	Sti-LVAT	0.181	0.162	0.164	0.044	3.808	< 0.001***
	Age			0.118	0.073	1.608	0.111
3	Sti-LVAT	0.217	0.189	0.153	0.044	3.499	0.001**
	Age			0.107	0.072	1.482	0.142
	NT-proBNP preprocedure			0.000	0.000	1.972	0.052
4	Sti-LVAT	0.239	0.203	0.149	0.043	3.432	0.001**
	Age			0.098	0.072	1.358	0.178
	NT-proBNP preprocedure			0.000	0.000	1.408	0.163
	QRSd preprocedure			0.035	0.023	1.559	0.123

*Sti-LVAT* stimulus to left ventricular activation time, *NT-pro-BNP* N-terminal pro-B type natriuretic peptide, *QRSd* QRS duration. *p values < 0.05, **p values < 0.01, ***p values < 0.001

### Complications and cardiac composite endpoints

A case with CHD in RVAP suffered from pocket hematoma 2-day post-procedure and received intermittent compression hemostasia. More transient RBB injuries were observed in LBBAP than and in RVSP and RVAP during the procedure (5/30 vs. 1/24 vs. 0, *p* = 0.014). Another case in LBBAP suffering from transient fever 3-day post-procedure was treated with antibiotic treatment for three days. No serious complications, including coronary artery injury, septal perforation and pocket infection, occurred during 12 months follow-up period. During 12 months follow-up, a case with diabetes in RVSP suffered from unstable angina and received percutaneous coronary intervention (PCI) in right coronary artery. Five patients in RVAP and one in RVSP were hospitalized with heart failure during the follow-up. No cardiovascular related death was observed in the three groups.

## Discussion

In this retrospective observational study, the main findings are as follows: (i) LBBAP was feasible with the same high success rate as RVSP and RVAP. (ii) LBBAP, characterized by an RBBB pattern, yielded the narrowest QRSd and the shortest Sti-LVAT among the three pacing methods and maintained physiological LV activation. (iii) Tip impedance, rather than bipolar impedance, was significantly lower in the LBBAP group than in the RVSP and RVAP groups during the 3-month post-procedure. LBBAP demonstrated stable pacing parameters during the 12-month follow-up regardless of lead polarity. (iiii) A significant reduction in LVEDD and NT-pro-BNP levels was achieved in the LBBAP group during the follow-up. (iiiii) The Sti-LVAT was a significant predictor of the left ventricular size at 12 months post-procedure.

Recently, LBBAP, as a near-physiological pacing innovation, demonstrated its feasibility in some previous observational studies [16, 17], with a high implant success rate (94.8–97.8%) owing to a fan network of LBBs and easily captured LBBs [18, 19]. In the present study, the implantation success rate in the LBBAP group was 90.91%, which was comparable to those of the RVSP (95.45%) and RVAP (100%) groups. In our study, cardiac CT was used to determine the optimal projection angle of the X-ray, which made the tip of 3830 lead truly perpendicular to the right ventricular septum. In addition, the optimal projection angle can accurately check the depth of lead tip into the ventricular septum to avoid the possible perforation by injecting 1–2 ml of contrast from the sheath. In our study, no ventricular septum perforation was observed during the procedure and 12-month follow-up. However, the average fluoroscopy time and X-ray dose were increased in the LBBAP group compared with those in the RVSP and RVAP groups due to the fixation of the LBB lead. Chen et al. [16] reported an average fluoroscopic exposure time of 9.15 min in the LBBP group, which was consistent with our study. The learning curve and experienced operators may be the main reasons for the differences.

QRSd is one of the most important indicator of correction of electromechanical dyssynchrony. Chen et al. reported the paced QRSd was 111.85 ± 10.77 ms in the LBB pacing group and 160.15 ± 15.04 ms in the RVP group (*p* < 0.001) [15]. The possible mechanisms are as follows. The LB-Purkinje conduction system fans out beneath the subendocardium of the left ventricular septum, which is directly used to synchronize left ventricular contraction and deliver near-physiological ventricular activation, and yield a short QRSd during LBBAP. In addition, it is a key reason that certain amount of right bundle branch fibres are recruited during LBBAP [20]. The magnitude of ventricular resynchronization by LBBAP compared favorably with the reduction of QRSd achieved by biventricular pacing [21]. However, RVSP and RVAP utilize the noncorresponding activation propagation pattern of the endomyocardium, which results in electromechanical dyssynchrony. In the present LBBAP group, the paced QRSd was much narrower than intrinsic QRSd (105.93 ± 15.85 ms vs. 122.67 ± 33.19 ms, *p* < 0.01), suggesting LBBAP produces a better ventricular electrical synchronization after procedure. The QRSd in LBBAP was the shortest in comparison with that in RVSP and RVAP (105.93±15.85 ms vs. 143.63±14.71 ms vs. 155.39±14.17 ms, *p* < 0.01), which is similar to the finding of Chen K et al. [15]. This indicated that the dyssynchrony with LBBAP was minimal comparing to RVSP and RVAP, which might be an important factor of good clinical outcomes. In addition to LBBAP, a pioneering investigation of LV septal sub-endocardial pacing was reported by Mafi-Rad et al. [22]. They found a shorter QRSd (144 ± 20 ms) when compared with that by RVP. However, the QRSd in Mafi-Rad M’s study was much longer than that by LBBAP (105.93 ± 15.85 ms) in our study. The possible explanation is the LBB was not directly captured as the pacing lead tip was in the deep mid-ventricular septum. Lan et al. reported that the 12-lead ECG of most LBBP patients maintained a normal QRS axis in their study [17]. However, in our study, the paced left axis deviation accounted for 43.33% in LBBAP patients due to the easier location and screwing in the left posterior branch area.

LVAT, another important electrophysiological index, indicates the rapidity of left ventricular lateral precordial myocardium depolarization. A constantly short Sti-LVAT was an essential criterion of the capture of the LBB during LBBAP [20]. Previous studies showed that the mean Sti-LVAT in LBBP ranged from 65.07 ± 8.58 ms to 73.78 ± 11.89 ms [16, 17]. In our study, the average Sti-LVAT in the LBBAP group was the shortest (72.80 ± 12.07 ms), followed by the RVSP (86.29 ± 8.71 ms) and RVAP (94.14 ± 10.14 ms) groups. A significant difference in the Sti-LVAT was observed among the three pacing methods (*p* < 0.001), which depended on whether the LB-Purkinje conduction system was captured. The Sti-LVAT was longer in RVSP and RVAP because activation propagation of the endomyocardium was slower than that of the natural LB-Purkinje conduction system. Thus, the Sti-LVAT may also be used as an important indicator of evaluating ventricular electromechanical dyssynchrony.

The nonphysiological ventricular activation pattern, such as in RVSP and RVAP, results in electromechanical dyssynchrony and adverse clinical outcomes [1, 2]. However, Lan S et al. observed a significant improvement in LVEF (57.08 ± 16.60% vs. 62.36 ± 12.20%, *P* < 0.001) and reduced LVEDD (52.27 ± 7.51 vs. 50.73 ± 6.71 mm, *P* < 0.001) by LBBAP after 1-year follow-up [17]. In this present study, improvements in LV size at 12 months post-procedure were significant in the LBBAP group as compared to baseline (*p* = 0.046). Meanwhile, a significant difference in ΔLVEDD from baseline to 12-month follow-up over time was noted among the three groups (LBBAP, RVSP, and RVAP) (−3.21 ± 4.06 mm vs. 0.77 ± 4.36 mm vs.3.00 ± 4.58 mm, *p* < 0.001). The significant change in LVEDD over time among the three groups could demonstrate the efficacy of LBBBAP.

Encouragingly, although the LBBAP group included more patients with LBBB and slightly higher NT-pro-BNP at baseline, we observed a significant reduction in the LVEDD and NT-pro-BNP values during LBBAP at 12 months post-procedure, suggesting that this more rapid ventricular activation improves LV function and reverses ventricular remodeling. Lan S et al. reported the patients with wide baseline QRS (> 120 ms) were observed to have significantly improved LVEF and reduced LVEDD [17], which was comparable to our study. In addition, despite having more patients with AF in the LBBAP group compared with the RVSP group and the RVAP group (23% vs. 8.3% vs. 5.3%), adverse LV remodeling in the other two groups did not occur in the LBBAP group, clearly suggesting this cohort to potentially benefit the most of resynchronization provided by LBBAP in a high percentage of ventricular pacing (> 75%).

In this study, we found that the Sti-LVAT was correlated with the LVEDD at 12 months post-procedure. Moreover, stepwise multiple regression analysis showed that the Sti-LVAT was an independent predictor of the LVEDD at 12 months post-procedure. All these findings indicated that the short Sti-LVAT during LBBAP represents near-physiological ventricular activation pattern, which translates into rapid ventricular activation and electromechanical synchronization, reverses ventricular remodeling. On the contrary, a wide Sti-LVAT means a prolonged ventricular activation time and a loss of physiological ventricular activation patterns, which might result in impaired left ventricular function.

Recently, more concerns have been focused on the pacing parameters of LBBAP. Previous studies by Chen et al. [15] and Chen et al. [16] demonstrated that the captured threshold, impedance and R-wave amplitude remained stable during the follow-up period. However, the polarity of the pacing parameters was not mentioned in most studies. Novelly, our study involved unipolar and bipolar pacing parameters. We found that the captured thresholds in LBBAP remained stable and low during the 12-month follow-up regardless of pacing polarity due to deep screwing-in and fixation of the pacing lead. During the procedure, captured thresholds in the LBBAP group were the highest among the three groups, which might result from more myocardial injury during LBBAP. Unlike HBP, LBBAP led to an incremental R-wave amplitude during follow-up, including unipolar and bipolar amplitudes, which might help prevent atrial oversensing and ventricular undersensing. In addition, our study demonstrated that unipolar rather than bipolar pacing impedance in the LBBAP group significantly decreased at 1 week post-procedure, and both unipolar and bipolar pacing impedances in the LBBAP group remained stable during the 12-month follow-up, similar to a previous study [17]. The underlying mechanism for the rapid decline in unipolar pacing impedance may be the alleviation of the edema of the myocardium around the tip of the lead and the tissue around the pacemaker post-procedure. Interesting, the polarity of pacing parameters was similar among the three groups, but not identical. In our view, the position of electrode ring is an important factor. In LBBAP, the ring was mostly in the interventricular septum and remained in contact with the myocardium.

In accordance with the previous study [16], a mild and transient increase in the cTnI levels was observed in LBBAP and RVSP post-procedure. However, LBBAP had a greater increase in the cTnI levels as compared to the other two groups due to deeper fixation and more attempts resulting in more myocardium injury during procedure. Encouragingly, this didn’t bring more complications and more adverse cardiac composite endpoints. On the contrary, RVAP depicted an increase trend in hospitalization of heart failure.

## Limitations

This was a single-center study with small sample sizes. The lack of randomization might lead to heterogeneous population and bias estimation on the results. The case crossover and the lack of the propensity score matching method might have resulted in bias estimation. In our study, the percentage of ventricular pacing was reduced due to the inclusion of the patients with SSS, which might have affected the final results. The comparisons between the patients with a higher percentage of ventricular pacing were more convincing. A total of 15 LBBB patients were included in our study. Subgroup analysis was not performed due to a small sample size. The 12-month follow-up was relatively short, and the long-term effects of electrophysiological and pacing parameters of LBBAP remain unclear at present. Prospective, randomized, long-term, large-scale and multicentered studies are required to determine the feasibility and effectiveness of LBBAP.

## Conclusions

LBBAP was feasible with the same high success rate as RVSP and RVAP. LBBAP demonstrated a stable and low captured threshold and unipolar pacing impedance and a high R-wave amplitude during the 12-month follow-up. Significantly improved LV remodeling at 12 months post-procedure was achieved through LBBAP. The Sti-LVAT was a significant predictor of LVEDD at 12 months post-procedure. LBBAP demonstrated its feasibility, effectiveness, safety and some beneficial electrophysiological characteristics during this mid- to long-term follow-up, and these results should be confirmed by further studies.

## Data Availability

The datasets used and/or analyzed during the current study can be available from the corresponding author on reasonable request.

## Abbreviations

LBBAP: Left bundle branch area pacing
RVSP: Right ventricular septal pacing
RVAP: Right ventricular apical pacing
Sti-LVAT: Stimulus to left ventricular activation time
LVEDD: Left ventricular end-diastolic dimension
HF: Heart failure
HBP: His bundle pacing
CT: Computerized tomography
RAO: Right anterior oblique
LAO: Left anterior oblique
ECG: Electrocardiogram
LVAT: Left ventricular activation time
AV: Atrioventricular
QRSd: QRS duration
LAD: Left atrial diameter
LVEF: Left ventricular ejection fraction
cTnI: Cardiac troponin I
NT-pro-BNP: N-terminal pro-B type natriuretic peptide
COI: The current of injury
PCI: Percutaneous coronary intervention
